# Effect of Obesity on the Respiratory Parameters in Children with Obstructive Sleep Apnea Syndrome

**DOI:** 10.3390/children10121874

**Published:** 2023-11-29

**Authors:** Carolina Caliendo, Rossella Femiano, Giuseppina Rosaria Umano, Stefano Martina, Ludovica Nucci, Letizia Perillo, Vincenzo Grassia

**Affiliations:** 1 Multidisciplinary Department of Medical-Surgical and Dental Specialties, University of Campania “Luigi Vanvitelli”, 80138 Naples, Italyletizia.perillo@unicampania.it (L.P.);; 2 Department of the Woman, the Child, and General and Specialized Surgery, University of Campania “Luigi Vanvitelli”, 80138 Naples, Italy; 3 Department of Medicine, Surgery and Dentistry “Scuola Medica Salernitana”, University of Salerno, 84123 Salerno, Italy; smartina@unisa.it

**Keywords:** obstructive sleep apnea syndrome (OSAS), Nocturnal Home Cardiorespiratory Polygraphy (PG), obesity, Saturation O_2_ (SaO_2_), Pediatric Sleep Questionnaire (PSQ)

## Abstract

The aim of the study was to investigate how obesity can influence sleep respiratory parameters in obstructive sleep apnea syndrome (OSAS) in children. Methods: The study analyzes 56 Caucasian children and adolescents aged 11 ± 2.79 years with a BMI > 5th percentiles and a PSQ value ≥ 0.33. Children were divided into Obesity Group (OG) with BMI ≥ 95th and Control Group (CG) with 5th < BMI > 95th percentile. All selected children underwent PG. Respiratory parameters AHI (Apnea–Hypopnea Index), SaO_2_ (Saturation of Oxygen), ODI (Oxygen Desaturation Index), and Nadir (the lowest value of SaO_2_ registered during PG) were extracted from the PG. AHI was used to divide the severity of OSAS into four levels: snoring (AHI ≤ 1), mild (AHI > 1 and ≤5), moderate (AHI > 5 and <10), and severe (AHI ≥ 10). Results: The comparison analysis between the OG and CG showed a statistical significance only for ODI (*p* = 0.02). A statistically significant correlation between BMI and AHI (r = 0.02), SaO_2_ (r = 0.01), and Nadir O_2_ (r = 0.02) was found. Conclusions: There was no strong correlation between obesity and OSAS, but a positive association was found between BMI and AHI severity.

## 1. Introduction

### 1.1. Obstructive Sleep Apnea Syndrome (OSAS)

The American Academy of Pediatrics (AAP) has defined Obstructive Sleep Apnea (OSA) as “a disorder of breathing during sleep characterized by prolonged partial upper airway obstruction and/or intermittent complete obstruction (obstructive apnea) that disrupts normal ventilation during sleep and normal sleep patterns” [1].

Many risk factors might contribute, through a reduction in or collapse of the upper airways, to the pathogenesis of OSAS. These risk factors include obesity, genetics, allergic rhinitis, craniofacial anomalies, adenoid and/or tonsil hypertrophy (such as Down, Prader–Willi, and Beckwith–Wiedmann), and inflammatory diseases [2].

About 2% of children have OSA, with boys being affected more frequently than females in association with the peak tonsil growth, due to the relative size of the Waldeyer ring lymphatic tissue [2]. A number of studies have found that after an adenotonsillectomy, children who are overweight or obese are more likely to continue having the disorder than children who have a normal weight, suggesting that obesity plays a major role in the pathophysiology of OSA [3,4].

### 1.2. Obesity and OSAS

Obesity in children is now recognized as a serious pediatric health concern. It is typically linked to a number of serious social and health issues (e.g., peer rejection, diabetes, and hypertension). Furthermore, data suggest that obstructive sleep apnea syndrome (OSAS) may be significantly impacted by juvenile obesity [5]. Numerous studies revealed that compared to children with normal weight, overweight or obese children had a 24–61% higher risk of developing OSAS [2,4]. A 10% increase in risk is linked to each body mass index (BMI) point above the 50th percentile. The relationship between body mass and OSAS in children seems to depend on a number of variables, including socioeconomic status, adenotonsillar hypertrophy, and ethnicity [6]. Obesity’s physiological effects on the respiratory system include the displacement of the diaphragm, the blunting of the central respiratory drive, a decreased chest wall compliance, an increased mass effect on the upper airway, and the fat deposition present in the pharyngeal muscles. These factors together increase the potential severity of OSAS [5]. In obese children, diffuse adiposity might lead to airway collapse during sleep, causing the onset and the increase in severity of OSAS, and an altered and short duration of sleep, might lead to an increase in appetite and body mass index (BMI), creating a vicious cycle. Indeed, it has been hypothesized that OSA contributes to obesity and vice versa [7].

### 1.3. Investigation of OSAS in Children

The diagnosis of pediatric OSAS is divided into several stages, the first one is a detailed collection of specific signs and symptoms, and amongst them, snoring is quite common (2–3% of children with snoring suffer from OSAS) [8]. Unfortunately, amongst children, a clear correlation cannot be easily found when it comes to comparing daytime signs and clinical assessment, and this could potentially lead to confusion [2]. The specialist should carry out specific and selective tests, also in the case of mild symptoms and risk factors, and the literature offers a wide variety of specific tests, leading to diverse predictability. With a range of sensitivity between 0.81 and 0.85 and a specificity of 0.87 for PSG-defined OSAS, the Pediatric Sleep Questionnaire (PSQ) is among the most reliable. It is unquestionably superior to other published questionnaires [9]. According to the most recent guidelines, it is a valid test for identifying children with OSAS, if their Apnea–Hypopnea Index (AHI) is greater than five [10,11,12]. The 22 symptom items on the test include a 2-item sleepiness subscale, a 6-item behavior subscale, a 9-item breathing subscale, and a 5-item another subscale that asks about weight, growth rate since birth, nocturnal enuresis, ability to wake up, and feeling tired in the morning. When eight or more of the twenty-two question items have a positive response, the sensitivity and specificity are both high [13]. Once the suspected diagnosis of OSAS has appeared through a first-level screening, it is necessary to evaluate sleep respiratory parameters with an examination such as Polysomnography (PSG). Polysomnography is the gold standard test to record parameters of the Apnea–Hypopnea Index (AHI), the Saturation of Oxygen (SaO_2_), the Oxygen Desaturation Index (ODI), and Nadir (the lowest value of SaO_2_ registered during polysomnography), in order to perform an OSAS diagnosis [2], to evaluate the severity, and to predict postoperative complications and the persistence of sleep breathing disorders after treatment. Due to the costs involved and the need for hospitalization, it unfortunately cannot be considered a routine examination [2]. The Nocturnal Home Cardiorespiratory Polygraphy (PG) might be used for OSA diagnosis as an alternative to PSG [14]. This is a portable, home-based device that measures heart rate, respiration, position, electrocardiogram (ECG), snoring, chest and abdominal wall movements, and pulse oximetry. However, PG detects apnea and/or hypopnea and respiratory effort but does not distinguish sleep from wakefulness and does not facilitate sleep staging. The affordability, the non-hospitalization requirement, takes into consideration that it can be performed in the patient’s normal bed in an accustomed environment, which allows for a more physiologically relevant study, especially in children, and is the advantage of this examination [15].

### 1.4. Literary Review

In the literature, several articles have examined the correlation between obesity and OSAS, and the conclusions drawn are often discordant. In 2012, the American Academy of Pediatrics (AAP) published an OSA guideline that recommends weight reduction in obese children, even though only some works have found and reported a significant association between weight change and increased polysomnography (PSG) parameters [9,11]. A review by Gulotta et al. [2] reported that few papers have investigated obesity as a risk factor and how it can influence AHI in children with OSAS; moreover, their results are very discordant [4,5,16,17]. Therefore, it is clear that obesity is a risk factor for OSAS in children, but the literature is unable to assess the degree to which this condition directly influences the respiratory parameters and the children’s daily lives.

### 1.5. Aim

The purpose of the study is to investigate how obesity can influence the AHI index and sleep respiratory parameters in OSAS pediatric patients. The primary endpoint is to evaluate how obesity can influence the AHI index and sleep respiratory parameters in OSAS pediatric patients using a non-invasive test as PG. The secondary endpoint is to investigate if PSQ is a valid tool to highlight the risk of OSAS patients as a preliminary screening.

## 2. Materials and Methods

### 2.1. Ethical Approval and Informed Consent

Approval for this observational epidemiological study was granted by the Institutional Review Board and Ethics Committee of the University of Campania “Luigi Vanvitelli” (n.776/2014). Signed informed consent from was obtained from the parents of all children involved in the study after a detailed explanation of the experimental protocol.

### 2.2. Sample Recruitment

Ninety-one consecutive Caucasian patients were gathered from the Program of Orthodontics, Multidisciplinary Department of Medical-Surgical and Dental Specialties and Department of the Woman, the Child, of General and Specialized Surgery, University of Campania “Luigi Vanvitelli”, Italy, between January 2019 and January 2020. Children were eligible if they were between 8 and 14 years of age, had a BMI > 5th percentile (a body mass index (BMI) > 5th for age and sex, according to reference charts of World Health Organization) [18] and a pathological score on a sleep questionnaire for OSA screening (PSQ value ≥ 0.33). Patients recruited in the sample had an age range starting from 8 years in order to eliminate the interference that the tonsillar peak growth may have [2].

All the observed patients were evaluated by an otolaryngologist who excluded any adeno-tonsillar problems or other types of conditions that could be the cause of OSA.

Patients with BMI ≤ 5th percentile, non-alimentary obesity, a medical history of acute or chronic cardiorespiratory or dimorphism, significant craniofacial abnormalities, neuromuscular disorders, or related craniofacial syndromes were excluded.

Of the 91 initial patients, 5 fulfilled one of the exclusion criteria (1 had a craniofacial syndrome, and 4 had hormonal abnormalities that led to obesity) and 18 did not wish to participate in the study.

Children who met the study criteria were divided into two groups: Control Group (CG), with 5th < BMI > 95th percentile, and Obesity Group (OG), with BMI ≥ 95th.

### 2.3. Clinical Examination

Weight was measured using a balance beam scale, and the child was undressed and was without shoes for the measurement. BMI was calculated as the weight in kilograms divided by the square of the height in meters (kg/m^2^), while an Harpenden stadiometer measured height. BMI percentile was calculated according to reference charts [18].

### 2.4. Pediatric Sleep Questionnaire

**Figure 1:**
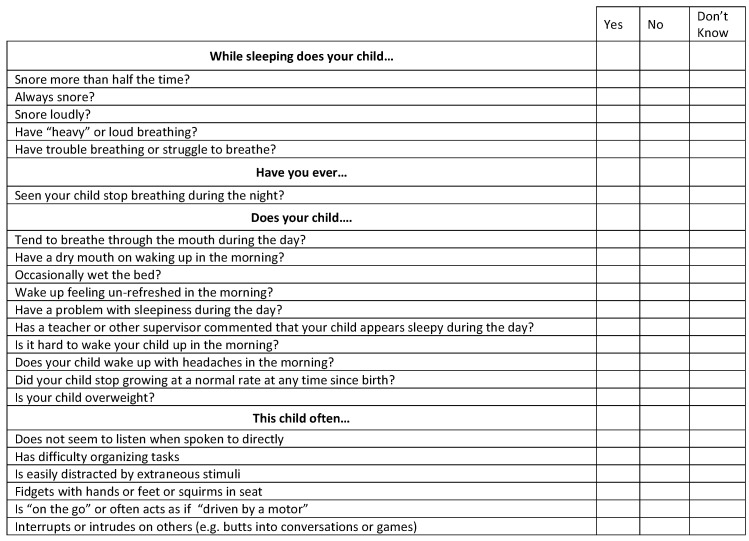
Pediatric Sleep Questionnaire (PSQ).

All the children’s parents filled out the Pediatric Sleep Questionnaire (PSQ) (Figure 1). The questionnaire was provided to parents after translating it from English to Italian, validated by a native language speaker. A cutoff value of 0.33 (of the 22 question items, 33% had positive responses) was used to assess the risk for OSAS [9]. We established a binary system in which the affirmative answers corresponded to 1, the negative corresponded to 0, and if the parents did not know, the response was missed.

### 2.5. Cardiorespiratory Polygraphy

**Figure 2:**
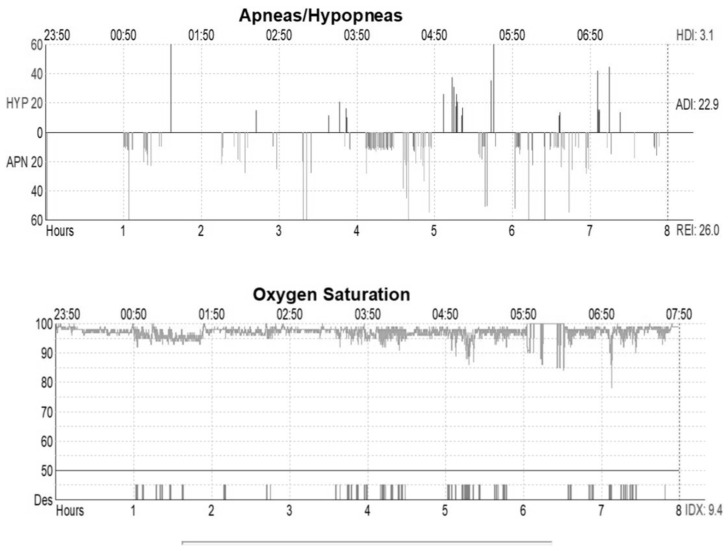
Nocturnal Home Cardiorespiratory Polygraphy report. ADI, apneas per hour of sleep; APN, apnea; Des, desaturation; HDI, hypopneas per hour of sleep; HYP, hypopnea; IDX, oxygen desaturation index; and REI, respiratory event index.

All selected children underwent a Nocturnal Home Cardiorespiratory Polygraphy (PG). A technician operator instructed the parents on the time when the child was to sleep and set the registration for nine hours. The following parameters were recorded simultaneously: airflow, snore, abdominal and chest effort parameters, electrocardiography (ECG), oximetry, and body position. The respiratory parameters AHI, SaO_2_, ODI, and Nadir were extracted from the report (Figure 2) and stored by creating an Excel file (Microsoft corporation, San Francisco, CA, USA). The same technician manually analyzed each sleep examination in order to prevent inter-observer variability. The samples were divided into four levels according to the OSAS severity using the AHI parameter: snoring with an AHI of ≤1; mild OSA with an AHI between >1 and ≤5; moderate OSA with an AHI between >5 and <10; and severe OSA with an AHI of ≥10. Twelve children and adolescents were excluded because of their inadequate signal quality/sleep time < 3.75 h or signal quality < 90%. The final sample included 56 patients.

### 2.6. Statistical Analysis

The study participants’ demographics and clinical characteristics were reported using appropriate means with standard deviations (SDs), medians with ranges, and frequencies with percentages. G*Power 3.1.9.6 for Mac OS was used to calculate the sample size, imposing three different settings: a single tail and an effect size of 0.5, an error probability ratio of 0.2, and a power level of 0.8. The clinical risk for OSA was indicated by a score of 0.33, which was then used to determine the sensitivity and specificity of the PSQ score to accurately identify OSA. Spearman Rho coefficients were used to evaluate the PSQ’s internal consistency. To compare the categorical variables between those at low and high risk, the Fisher exact test was employed to examine any differences. Spearman Rho coefficients were utilized to evaluate correlations among the physical examination findings, PSQ items (within the total PSQ score), AHI, SaO_2_, Nadir O_2_, and PSG parameters and the outcomes. A χ^2^ test was used to compare the univariate analysis of the categorical data, and logistic regression was used to perform multiple analyses that included all risk factors with *p* ≤ 0.05. Odds ratios (ORs) with 95% confidence intervals were provided for each statistically significant factor. SAS version 9.4 was used for all analyses, and a two-sided *p*-value of less than 0.05 was deemed statistically significant. Student’s *t*-test was used for paired data to find any systematic error, and Dahlberg’s D was computed to quantify the measurement error. The analyses were performed with the R version 4.3.2 “Eye Holes”software environment for statistical computing. The level of significance was set at *p* ≤ _0.05 [19].

## 3. Results

In this study, 56 Caucasian children and adolescents (30 males and 26 females, aged 11 ± 2.79 years) were enrolled. The demographic and anthropometric characteristics of the sample are shown in Table 1. Based on the BMI, in the sample, 41.1% of children were in the Control Group (5th < BMI > 95th), while 58.9% were in the Obesity Group (BMI 95th). The two groups differed significantly in Z-score BMI, with a value of 0.63 for the Control Group and a value of 3.77 for the Obesity Group (*p* < 0.00). After an PG evaluation of 56 patients, 14.3% of cases exhibited snoring, 33.9% mild OSAS, 32.2% moderate OSAS, and 19.6% severe OSAS. Regarding OSAS parameters, the two groups differed significantly only in the ODI, with a value of 9.67 and 3.72, respectively (*p* = 0.02) (Table 2).

### 3.1. Comparison Analysis of PSQ between the Obesity Group and the Control Group

The comparison analysis of the PSQ between the two groups showed a higher value of the questionnaire for the Obesity Group than that for the Control Group, but the difference was not significant (*p* = 0.15) (Table 2).

### 3.2. Correlation Analysis of PSQ with Z-Score BMI and Respiratory Parameters

The correlation analysis of the PSQ shows no significant result with Z-score BMI (r = 0.02). In the same way, our analysis of the correlation between PSQ and the severity of OSAS (AHI r = 0.02) and other respiratory parameters (SaO_2_ r = 0.01, ODI r = 0.03, Nadir O_2_ r = 0.02) showed no significance (Table 3).

### 3.3. Correlation Analysis between BMI and Respiratory Parameters

**Figure 3:**
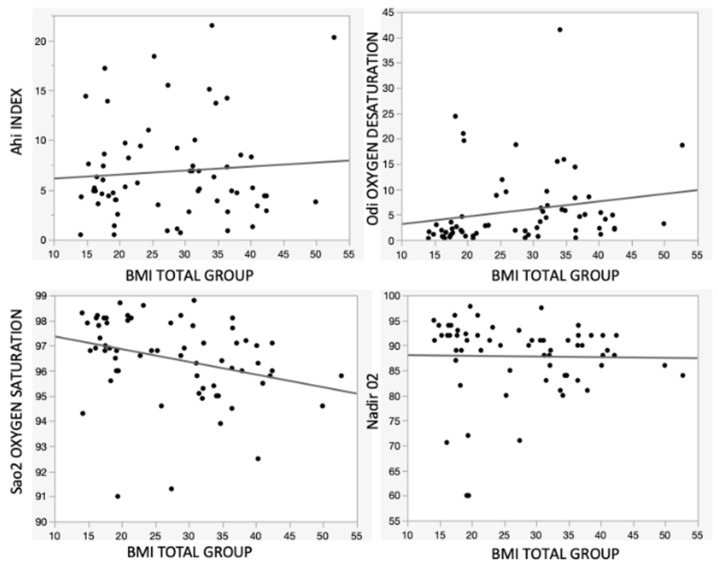
Linear regression line illustrating the relation between Z-score BMI and respiratory parameter.

The linear regression model was used to investigate the changes in respiratory parameters according to the increase in BMI. The graphic showed a statistically significant direct correlation between the AHI and the Z-score BMI. Moreover, the slope of the line showed that an increase in Z-score BMI equaled an average decrease in the SaO_2_, and it was statistically significant. The linear regression analysis with the ODI illustrated that it did not show any correlation with the Z-score BMI. Finally, it was found that there was a statistically significant correlation between the Z-score BMI and Nadir O_2_ (Figure 3 and Table 4).

### 3.4. Distribution of the Entity of OSAS and SaO_2_

**Figure 4:**
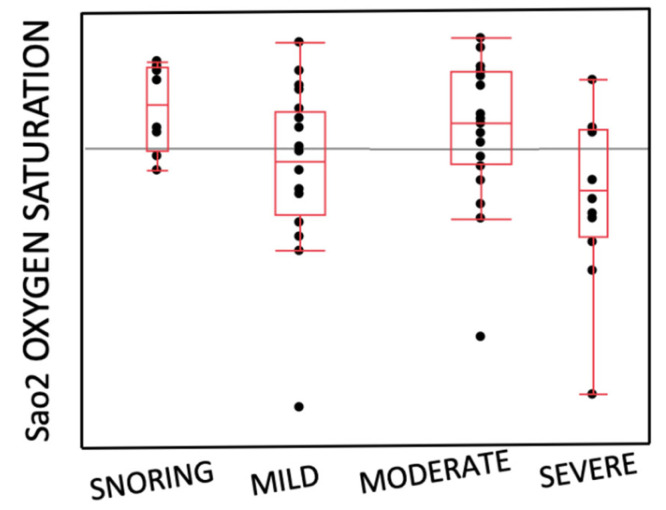
Box and whiskers plot illustrating the relation between the entity of OSAS (x: AHI) and oxygen saturation (y: SaO_2_).

The box and whiskers plot (Figure 4) reported the SaO_2_ distribution in the different OSAS severity categories. The graph presented a horizontal line corresponding to a saturation of 97, the average oxygen saturation value in a healthy subject. As shown in the diagram, the box and the corresponding median of snoring was positioned above the value of 97, and its lower dispersion index was just below the normal value. The mild OSAS reported a SaO_2_ below the median, but very close to the normal oxygen saturation value. Unlike the mild form, in the severe OSAS, the saturation of oxygen was reported to be below the median, but with a lower dispersion index far below the value of 97. Finally, in the moderate form of OSAS, the median was placed above the value 97, but its dispersion index was very far from the normal saturation.

## 4. Discussion

The aim of the study was to investigate how obesity might influence the AHI index and sleep respiratory parameters in children with OSAS.

Andersen investigated the impact of obesity treatment on OSA in children and adolescents, indicating that obesity treatment should be considered among the first-line treatments. He reported an association between BMI reduction and AHI improvement after approximately six months of treatment [20].

It has been reported that 1 kg/m^2^ beyond the mean BMI for age and gender can increase the risk of OSA by 12% [7], and children older than 12 years old show an increased risk of OSAS and is associated with them being overweight and having obesity in their adulthood [3,21].

We hypothesized that obese children might have a greater risk of being affected by OSAS than the average population, and a positive correlation was found between the degree of obesity and the severity of OSAS. The comparison between the Obesity Group and the Control Group showed a statistically significant difference in age, BMI and Z-score BMI.

Andersen, Arens, and Xu reported a statistically significant difference in the AHI between the two groups [4,17,22].

Conversely, in our study, the Obese Group exhibited a higher value of the AHI, but its difference with the Control Group’s AHI value was not statistically significant. This finding is similar to the study by Su et al., in which a very large sample of children with OSAS with and without obesity was analyzed, reporting an AHI value that was not statistically significant [23]. Then, the comparative analysis of the different respiratory parameters was conducted, and we reported a lower oxygen saturation, a higher ODI value, and a lower Nadir O_2_ value for the Obesity Group than the Control Group, but only the ODI showed a statistically significant difference. The study of Andersen et al. was very similar to ours, and they reported a non-statistically significant difference in both SaO_2_ and ODI [4]. Conversely, our study showed a statistically significant ODI; this means that the obese group presented several episodes of desaturation below 4%, indicating the importance of monitoring saturation and its decrease in obese children. Furthermore, we conducted a correlation analysis to verify the value of the respiratory parameters, according to the increase in BMI.

Our statistical analysis showed a linear statistically significant decrease in SaO_2_ values and a constant correlation value of Nadir O_2_ with increasing BMI, which was statistically significant, while the regression analysis reported a statistically significant worsening of the AHI with an increase in the Z-score BMI. The difference in the results between the comparative and the correlation analyses could be due to a bias exhibited by the comparative analysis for the two groups, which differs greatly with the size of groups, while the correlation analysis does not depend on the size of groups.

The linear analysis between BMI and ODI showed a positive correlation but was not statistically significant; this result may also be explained by the presence of a non-large sample size that was enough to detect the significance of all parameters.

In the literature, the studies of Xu et al., Koehler et al., and Andersen et al. [4,5,22] have reported similar results. In the study of Xu [22], obesity was positively related to the number of obstructive apnea/hypopnea events and was inversely related to Nadir in obese children. The correlation analysis of Kohler et al. indicated that children with a higher BMI-z score demonstrated a significantly higher number of obstructive events during sleep and a lower Nadir O_2_ [5]. Andersen et al. showed that an increase in the BMI SDS of one unit equaled an average increase in the AHI of 57% [4]. However, it is challenging to directly compare this study with other studies because respiratory events included in the AHI are not always defined consistently, and there is a disagreement over the AHI cutoff value that defines OSA. The term “obesity” is also defined differently in different studies.

Carroll et al. issued a warning, stating that diagnosing OSAS with clinical evaluation alone was inadequate [9,11], and a meta-analysis published in 2004 by Brietzke et al. reached the same conclusion [23]. Following these findings, a number of instruments, such as clinical scoring scales and questionnaires, have been developed to improve the screening of these patients [8]. Compared to other published questionnaires, the Pediatric Sleep Questionnaire (PSQ) has a specificity of 0.87 and a sensitivity range of 0.81 to 0.85 for PSG-defined OSAS, making it one of the most reliable instruments. A study by Umano et al. reported a statistically significant correlation between the PSQ and the AHI, demonstrating the validity of the questionnaire as a valid tool for the screening and diagnosis of OSAS. Moreover, ROC curve analysis showed that the PSQ exhibited a good accuracy for moderate-to-severe OSA diagnosis, with a cutoff value of 0.65 in obese children [12]. Our study conducted a comparative analysis of the PSQ between the two groups and the PSQ with respiratory parameters. The PSQ values were higher in the Obese Group as they reflected a higher symptomatic stage reported by patients and parents, but not a significantly different value for the data was reported. It is important to highlight that the investigation through the questionnaire could potentially be limited by the fact that it is not filled by the children themselves but by their parents, who do not always spend the night sleeping with their kids and have little knowledge of their actual sleeping behavior [7]. This could obviously generate a bias in both groups. Furthermore, this result may be explained by the presence of a non-large sample size that was enough to detect the significance of the PSQ. In any case, the PSQ should be considered a valid tool of initial screening for parents, teachers, and clinicians to focus their attention on the behaviors and habits of a child and to prevent the risk of more serious consequences. Therefore, to obtain the maximum output using the PSQ as a screening tool, all the children with a score > 0.33 should be subjected to sleep nocturnal monitoring, like PG, to obtain an adequate diagnosis of OSAS and an adequate evaluation of the respiratory parameters of patients. We unfortunately were unable to perform a full PG; thus, the information about arousals and sleep architecture was not acquired, and this could potentially result in an underestimation of the respiratory parameters. Another analysis performed in our research evaluated the distribution of SaO_2_ amongst the various OSAS severity classes. In snoring, subjects reported an average SaO_2_ value of above 97, very close to the normal range. Furthermore, we found that in mild and moderate OSAS, the distribution of SaO_2_ values did not follow a specific trend, indicating the need to provide maximum attention to monitoring the parameter during the diagnosis procedure. Contrarily, in subjects with severe OSAS, the SaO_2_ values were well below the normal threshold. However, these data must always be compared with Nadir O_2_. If this parameter maintains optimal values, the health risks are contained, and on the other hand, if its values are reduced, an extremely dangerous situation would arise.

OSAS in children is a condition with multifactorial etiology. The coexistence of daytime symptoms regarding a child’s academic progress, attention, behavior or emotion regulation, sustained attention, selective attention, and alertness with nocturnal symptoms like snoring or arousal should alert clinicians [24,25,26,27]. Undiagnosed OSAS can lead to growth impairment, cardiovascular problems, and neurobehavioral issues.

Therefore, it is important to identify risk factors for OSAS with valid tools like PSQ and PG to develop better strategies for managing OSAS.

## 5. Conclusions

The results of our study have shown that there was no strong correlation between obesity and, OSAS but a positive association was found between the BMI and the AHI severity. No statistically significant correlation was found between the results of the PSQ for the Obesity Group and the Control Group in terms of respiratory parameters, except for the ODI.

## Figures and Tables

**Table 1:** Demographic and anthropometric characteristics. SD, standard deviation; BMI, body mass index.

	Obesity Group	Control Group	*p*
Subjects	33	23	0.88
Male	19	11	0.47
Female	14	12	0.47
Mean Age ± Sd (years)	10.47 ± 2.93	8.72 ± 2.25	0.02
BMI ± Sd (kg/m^2^)	34.95 ± 7.27	17.69 ± 2.31	<0.00
Z-score BMI	3.77 ± 0.87	0.63 ± 0.95	<0.00

**Table 2:** OSAS parameters and PSQ value of both groups.

	Obesity Group	Control Group	*p*
Osas Severity			0.97
Snoring (14.3%)	5 (15.2%)	3 (13%)	
Mild (33.9%)	11 (33.3%)	8 (34.7%)	
Moderate (32.2%)	10 (30.3%)	8 (34.7%)	
Severe (19.6%)	7 (21.2%)	4 (17.6%)	
AHI	7.04 ± 5.80	6.23 ± 4.31	0.46
ODI	9.67 ± 10.30	3.72 ± 6.29	0.02
Nadir O_2_	86.45 ± 6.76	88.94 ± 9.40	0.36
SaO_2_	92.67 ± 1.27	96.2 ± 1.57	0.38
PSQ	0.53 ± 0.13	0.47 ± 0.15	0.15

**Table 3:** Correlation analysis of PSQ with Z-score BMI and respiratory parameters.

	r^2^	*p*
Z-score BMI	0.02	0.87
AHI	0.02	0.76
SaO_2_	0.01	0.61
ODI	0.03	0.81
Nadir O_2_	0.02	0.66

**Table 4:** Correlation analysis of Z-score BMI with respiratory parameters.

	r^2^	*p*
AHI	0.01	<0.00
SaO_2_	0.09	<0.00
ODI	0.04	0.56
NADIR O_2_	0.04	<0.00

## Data Availability

The data presented in this study are available on request from the corresponding author. The data are not publicly available due to their containing information that could compromise the privacy of research participants.
